# LLVM-based Hybrid Fuzzing with LibKluzzer (Competition Contribution)

**DOI:** 10.1007/978-3-030-45234-6_29

**Published:** 2020-03-13

**Authors:** Hoang M. Le

**Affiliations:** 8 University of Paderborn, Paderborn, Germany; 9 ICREA, Open University of Catalonia, Barcelona, Spain; Insitute of Computer Science, University of Bremen, Bremen, Germany

**Keywords:** Hybrid Fuzzing, Coverage-guided Fuzzing, Symbolic Execution, LLVM

## Abstract

LibKluzzer is a novel implementation of hybrid fuzzing, which combines the strengths of coverage-guided fuzzing and dynamic symbolic execution (a.k.a. whitebox fuzzing). While coverage-guided fuzzing can discover new execution paths at nearly native speed, whitebox fuzzing is capable of getting through complex branch conditions. In contrast to existing hybrid fuzzers, that operate directly on binaries, LibKluzzer leverages the LLVM compiler framework to work at the source code level. It employs LibFuzzer as the coverage-guided fuzzing component and KLUZZER, an extension of KLEE, as the whitebox fuzzing component.

## 1 Test Generation Approach

LibKluzzer is based on hybrid fuzzing which tries to combine the strengths of coverage-guided fuzzing and whitebox fuzzing. Most existing advanced hybrid fuzzers, e.g. [6,7,8], employ coverage-guided fuzzing as the main search algorithm and only apply whitebox fuzzing selectively on the most promising inputs. While such advanced approach is also being under development and evaluation for LibKluzzer, for simplicity and given the short time frame available for adapting to Test-Comp, the participating version of LibKluzzer combines coverage-guided fuzzing and whitebox fuzzing in a very simple way. Without any intrinsic integration, multiple instances of coverage-guided fuzzing and whitebox fuzzing are scripted to run in parallel in their own OS process. They operate on a common corpus to enable sharing the individual progresses. Each instance keeps an in-memory set of inputs it has generated, together with the code coverage achieved so far. Whenever an instance discovers an input that covers new code, it writes this input as a file to the common corpus. The corpus is scanned periodically by the instances to check for newly added files. Despite of (or thanks to) its simplity, LibKluzzer managed to perform very well in Test-Comp 2020.

## 2 Software Architecture

Two major components of LibKluzzer are LibFuzzer [1] for coverage-guided fuzzing and KLUZZER [5] for whitebox fuzzing. As mentioned earlier, KLUZZER is an extension of KLEE [2]. While it uses most of the KLEE infrastructure including the underlying SMT solver STP [3], KLUZZER provides several signicant enhancements that make it more suitable for hybrid fuzzing (see [5] for more details). For Test-Comp, both LibFuzzer and KLUZZER have been extended to support its specific requirements. The extension involves writing test cases in XML format, glue logic to convert the random byte array needed for the fuzzers into a sequence of calls to *nondet* functions, and implementing a fuzzing target as described later.

**Workflow** First, the C program under test undergoes a set of source-to-source program transformations to enable *in-process* coverage-guided fuzzing. The transformed program is then compiled using Clang to create an LLVM bitcode file and an executable. The compilation involves, among others, code coverage instrumentation and linking with LibFuzzer. Finally, the LLVM bitcode file is fed to KLUZZER to perform whitebox fuzzing, while the executable is started in two instances to perform coverage-guided fuzzing. These three fuzzing instances run concurrently until terminated by the Test-Comp BenchExec runner due to time limit exceeded. They share generated inputs via a common corpus of files as mentioned earlier and write XML test cases to the test suite on-the-fly.

**Transformations for in-process fuzzing** While the main components of LibKluzzer are implemented in C++, the program transformations, that are required to enable *in-process* coverage-guided fuzzing, consist of a set of Bash and Python scripts. This form of fuzzing is much faster than traditional out-of-process fuzzing, which forks a new process for each execution of the *main* function, but requires the global state of the fuzzing target to remain largely unchanged or to be resetted between executions. The transformations esssentially perform the following steps for each benchmark:

1. rename the existing *main* function to *FuzzMe*;
2. identify and duplicate global variables;
3. insert additional functions: *FuzzerSaveCtx* to capture the initial global state into the duplicated variables and *FuzzerRestoreCtx* to restore this state before each new execution of the *FuzzMe* function;
4. redirect calls to *exit* and *abort* to custom functions to prevent unwanted early exit from the fuzzing loop.

The current script-based implementation of these transformations is very fragile and might not work out-of-the-box for non-Test-Comp benchmarks. The next version of LibKluzzer will replace these with proper Clang-based source-to-source transformations.

```
int nondet_int() {
  int Value = 0;
  if (Used + 4 <= Size) {
    memcpy(&Value, Data + Used, 4);
    Used += 4;
  }
  return Value;
}
```

```
int LLVMFuzzerTestOneInput(
    uint8_t *Data, size_t Size) {
  FuzzerRestoreCtx();
  MakeGlobalCopy(Data, Size);
  Used = 0;
  FuzzMe();
}
```

**Fig. 1.** Implementation of *nondet* functions and fuzzing target for Test-Comp

**Test-Comp fuzzing target and *nondet* functions** Both KLUZZER and LibFuzzer require the definition of a fuzzing target, i.e. an implementation of the declared *LLVMFuzzerTestOneInput* function. The *main* function provided by the fuzzers will repeatedly call *LLVMFuzzerTestOneInput* with fuzz inputs in a loop to perform fuzzing. Each fuzz input consists of an array of random bytes and its size. Fig. 1 shows a conceptual implementation of *LLVMFuzzerTestOneInput* on the right hand side. First, the initial global execution state is restored. Then, the given fuzz input is copied into a global array and the number of bytes already consumed for fuzzing is set to zero; Finally, *FuzzMe* is invoked. During its execution, each time a *nondet* function is called to provide input, a corresponding number of bytes from the global byte array will be consumed to create the requested value, as exemplarily shown on the left hand side of Fig. 1 for *int*. With this conversion from random bytes, no changes are needed in the core algorithms of KLUZZER and LibFuzzer for Test-Comp.

## 3 Strengths and Weaknesses

The main strength of LibKluzzer lies in achieving high code coverage as demonstrated by winning the branch coverage category of Test-Comp. Multiple factors contribute to this success including the extremely high throughput of in-process coverage-guided fuzzing implemented by LibFuzzer and the use of generational search in KLUZZER, a coverage-maximizing search heuristic for dynamic symbolic execution/whitebox fuzzing first proposed by SAGE [4]. The individual contribution of each single component is to be analyzed more thoroughly in a further detailed study.

The main conceptual weakness of LibKluzzer is that the same coverage-maximizing search strategy is used for reaching error calls. It is a big surprise that LibKluzzer has still achieved the second place in the corresponding category. We expect that adapting the search heuristics of both LibFuzzer and KLUZZER to be directed by the distance to the location of error calls should improve the performance significantly.

Especially, the big ECA benchmarks have proven to be problematic for both LibFuzzer and KLUZZER and hence also for LibKluzzer. The sequence of *nondet* values required to reach the error calls is very specific and nearly impossible to find with coverage-guided fuzzing, while KLUZZER suffers from path explosion.

In addition to error-directed search, path/state merging might be required to efficiently deal with these benchmarks.

A further weakness is that LibKluzzer makes little effort on minimizing the test suite with respect to both the size of the test suite and the size of each test case. Too many redundant test cases might cause the validator to timeout. Furthermore, some produced test cases are too big hitting a corner case in the validator and forcing it to exceed the given memory limit. In these cases, the validator crashes prematurely, leaving the remaining test cases uncounted.

## 4 Tool Setup and Configuration

**Installation** The LibKluzzer archive submitted to Test-Comp 2020 (version 0.6) can be downloaded from https://gitlab.com/sosy-lab/test-comp/archives-2020/blob/testcomp20/2020/libkluzzer.zip. After unpacking, the main executable script LibKluzzer can be found in the *bin* folder.

**Configuration** The main script has been configured to reflect the resource restrictions of Test-Comp 2020. LibKluzzer treats every benchmark as 64-bit and always tries to maximize code coverage, and thus is agnostic to the property and architecture specification. The only meaningful parameter is the path to the source code file of the benchmark.

**Participation** LibKluzzer participates in both available categories of Test-Comp 2020: *Finding Bugs* and *Code Coverage*.

## 5 Software Project and Contributors

LibKluzzer and KLUZZER are being developed by the author at University of Bremen, Germany. This research and development are supported by the Central Research Development Fund, University of Bremen, Germany within the project SYMVIR. The source code of LibKluzzer will be made available at https://github.com/hoangmle/LibKluzzerTestComp2020Submission. Much of the credits should go to the respective development teams of LibFuzzer and KLEE, which lay the foundation for LibKluzzer.
